# Ubiquitin‐specific protease 22 promotes tumorigenesis and progression by an FKBP12/mTORC1/autophagy positive feedback loop in hepatocellular carcinoma

**DOI:** 10.1002/mco2.439

**Published:** 2023-12-01

**Authors:** Qianwei Ye, Wei Zhou, Shengjun Xu, Qingyang Que, Qifan Zhan, Lincheng Zhang, Shusen Zheng, Sunbin Ling, Xiao Xu

**Affiliations:** ^1^ Department of General Surgery Hangzhou First People's Hospital Hangzhou China; ^2^ Zhejiang University School of Medicine Hangzhou China; ^3^ Key Laboratory of Integrated Oncology and Intelligent Medicine of Zhejiang Province Hangzhou China; ^4^ NHC Key Laboratory of Combined Multi‐Organ Transplantation Hangzhou China; ^5^ State Key Laboratory for Diagnosis and Treatment of Infectious Diseases, The First Affiliated Hospital, School of Medicine Zhejiang University Hangzhou China

**Keywords:** autophagy, FK506‐binding protein 12 (FKBP12), hepatocellular carcinoma (HCC), mammalian target of rapamycin complex 1 (mTORC1), tumorigenesis, ubiquitin‐specific protease 22 (USP22)

## Abstract

Ubiquitin‐specific protease 22 (USP22) has been identified as a potential marker for cancer stem cells in hepatocellular carcinoma (HCC). It can promote HCC stemness, which is considered a driver of tumorigenesis. Here, we sought to determine the role of USP22 in tumorigenesis, elucidate its underlying mechanism, and explore its therapeutic significance in HCC. As a result, we found that tissue‐specific Usp22 overexpression accelerated tumorigenesis, whereas Usp22 ablation decelerated it in a c‐Myc/NRasGV12‐induced HCC mouse model and that the mammalian target of rapamycin complex 1 (mTORC1) pathway was activated downstream. USP22 overexpression resulted in increased tumorigenic properties that were reversed by rapamycin in vitro and in vivo. In addition, USP22 activated mTORC1 by deubiquitinating FK506‐binding protein 12 (FKBP12) and activated mTORC1, in turn, further stabilizing USP22 by inhibiting autophagic degradation. Clinically, HCC patients with high USP22 expression tend to benefit from mTOR inhibitors after liver transplantation (LT). Our results revealed that USP22 promoted tumorigenesis and progression via an FKBP12/mTORC1/autophagy positive feedback loop in HCC. Clinically, USP22 may be an effective biomarker for selecting eligible recipients with HCC for anti‐mTOR‐based therapy after LT.

## Graphical abstract

USP22 promoted tumorigenesis and progression by an FKBP12/mTORC1/autophagy positive feedback loop in HCC. USP22 overexpression increased mTORC1 activity and sensitized HCC toward rapamycin. Mechanistically, USP22 interacted with FKBP12 to activate mTORC1 pathway through deubiquitylation. In addition, increased mTORC1 activity further stabilized USP22 by inhibiting autophagic degradation.

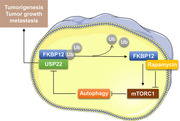

## 1 INTRODUCTION

Liver cancer ranked third among cancer‐related deaths in 2020 worldwide,^1^ and in China, it has already become the leading cause of cancer mortality in adult men under 60 years of age. Till today, surgery remains the most effective treatment for liver cancer. However, many patients with liver cancer are diagnosed at advanced stages and have no chance for surgery, leading to a high mortality rate.^2^

Ubiquitin‐specific protease 22 (USP22), a highly conserved mammalian ubiquitin hydrolase, is a significant component of the Spt‐Ada‐Gcn5 acetyltransferase complex (SAGA).^3^ USP22 participates in cell cycle regulation^4^, ^5^, ^6^ and telomere maintenance,^7^, ^8^ suggesting that it is closely associated with tumor proliferation disorders. In addition, cancer stem cells (CSCs) are considered carcinogenesis drivers.^9^, ^10^ USP22 has been identified as a potential CSC marker,^11^, ^12^, ^13^ implying its role in carcinogenesis. In hepatocellular carcinoma (HCC), we previously discovered that when the tumor protein p53 (TP53) was inactivated, USP22 was upregulated, promoting hypoxia‐induced HCC stemness.^13^ Therefore, the association between USP22 expression and tumorigenesis warrants further investigation. Interestingly, USP22 deletion, rather than overexpression, has been shown to drive tumorigenesis in colorectal cancer and myeloid leukemia.^14^, ^15^ Therefore, the role of USP22 in hepatocarcinogenesis warrants further investigation.

Recent studies have focused on identifying USP22 substrates, which have helped shed light on the involvement of USP22 in cancer initiation and progression.^3^ Protein modifications such as ubiquitination and deubiquitination are critical for protein stability and function. USP22 is the only SAGA subunit that can directly bind to oncogenic signals in the absence of SAGA.^4^, ^16^ Furthermore, our findings demonstrated that USP22 might operate as a target of hypoxia‐inducible factor 1α and increase glycolysis in HCC cells.^13^

Here, we sought to determine the role of USP22 in hepatocarcinogenesis and provide evidence for the association between USP22 and tumorigenesis in HCC. Mechanically, we attempted to identify the downstream target genes and novel subunits of USP22 to shed light on the molecular regulatory mechanisms underlying this phenomenon. Clinically, we explored the effect of USP22 on the prognosis of patients with HCC after liver transplantation (LT) to provide advice for clinical therapy.

## 2 RESULTS

### 2.1 Overexpression of Usp22 accelerates c‐Myc/NRasGV12‐induced HCC in mice

**FIGURE 1:**
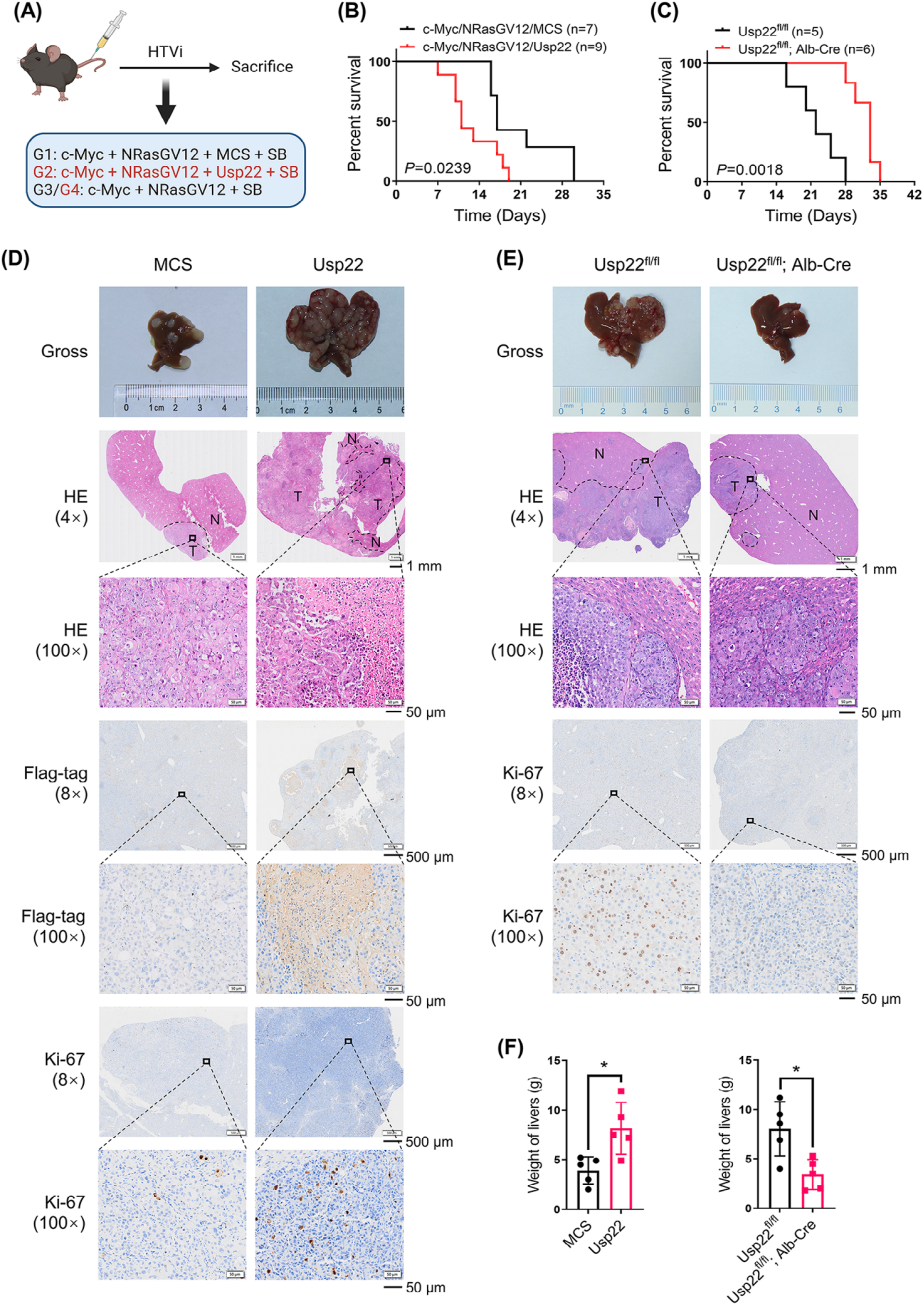
Usp22 accelerates c‐Myc/NrasGV12‐induced hepatocarcinogenesis. (A) Study design (created with BioRender.com). C57BL/6J mice were grouped as G1, G2, G3, and G4. (B) Survival curves of G1 (*n* = 7) and G2 (*n* = 9). Usp22 overexpression shortened the survival time of mice (*p* = 0.0239). (C) Survival curves of G3 (*n* = 5) and G4 (*n* = 6). Usp22 ablation prolongs the survival time of mice (*p* = 0.0018). (D) Representative images of gross liver specimens, hematoxylin–eosin (HE), Flag‐tag, and Ki‐67 staining in the G1 and G2 groups. (E) Representative images of gross liver specimens, HE, and Ki‐67 staining in the G3 and G4 groups. (F) The weight of livers in each group (^*^ *p* < 0.05).

A well‐established mouse HCC model induced by hydrodynamic co‐expression of c‐Myc and NRasGV12 oncogenes (c‐Myc/NRasGV12) in the liver was used to determine the role of Usp22 in hepatotumorigenesis (Figure 1A). Briefly, mice were hydrodynamically injected with c‐Myc, NRasGV12, and Flag‐tagged Usp22 plasmids (c‐Myc/NrasGV12/Usp22) as well as Sleeping Beauty (SB) transposon. The Usp22 plasmid was replaced with an empty multiple cloning site (MCS) vector in the control group (c‐Myc/NRasGV12/MCS). Noticeably, overexpression of Usp22 reduced the survival time of the c‐Myc/NRasGV12 mouse model of HCC (Figure 1B). The median survival time was 11 days for c‐Myc/NRasGV12/Usp22‐injected mice (*n* = 9) and 17 days for c‐Myc/NRasGV12/MCS‐injected mice (*n* = 7) (*p* = 0.0239). Additionally, liver‐specific Usp22 knockout mice were used to explore the effects of Usp22 ablation. As expected, Usp22^fl/fl^; Alb‐Cre mice (*n* = 6) survived significantly longer than Usp22^fl/fl^ mice (*n* = 5) after c‐Myc/NRasGV12 injection (33 days vs. 22.5 days, *p* = 0.0018) (Figure 1C). The experiment was repeated, and 2 weeks after injection, all mice were euthanized and dissected to observe the livers. The results showed that overexpression of Usp22 accelerated tumorigenesis, whereas Usp22 ablation decelerated it in the mouse HCC model (Figure 1D,E). Hematoxylin–eosin staining revealed that HCC developed successfully in all mice. Immunohistochemistry (IHC) of Flag‐tag confirmed ectopic injection of Usp22 in the liver, and Ki‐67 demonstrated the cell proliferation status in each group (Figure 1D,E). The weight of the livers, percentage of tumor area, and positive Ki‐67 results are shown in Figures 1F and S1. These results further validated that the tumors with Usp22 overexpression were significantly larger and more aggressive than those in the control group, whereas Usp22 ablation diminished this effect.

### 2.2 USP22 activates the mTORC1 signaling pathway

**FIGURE 2:**
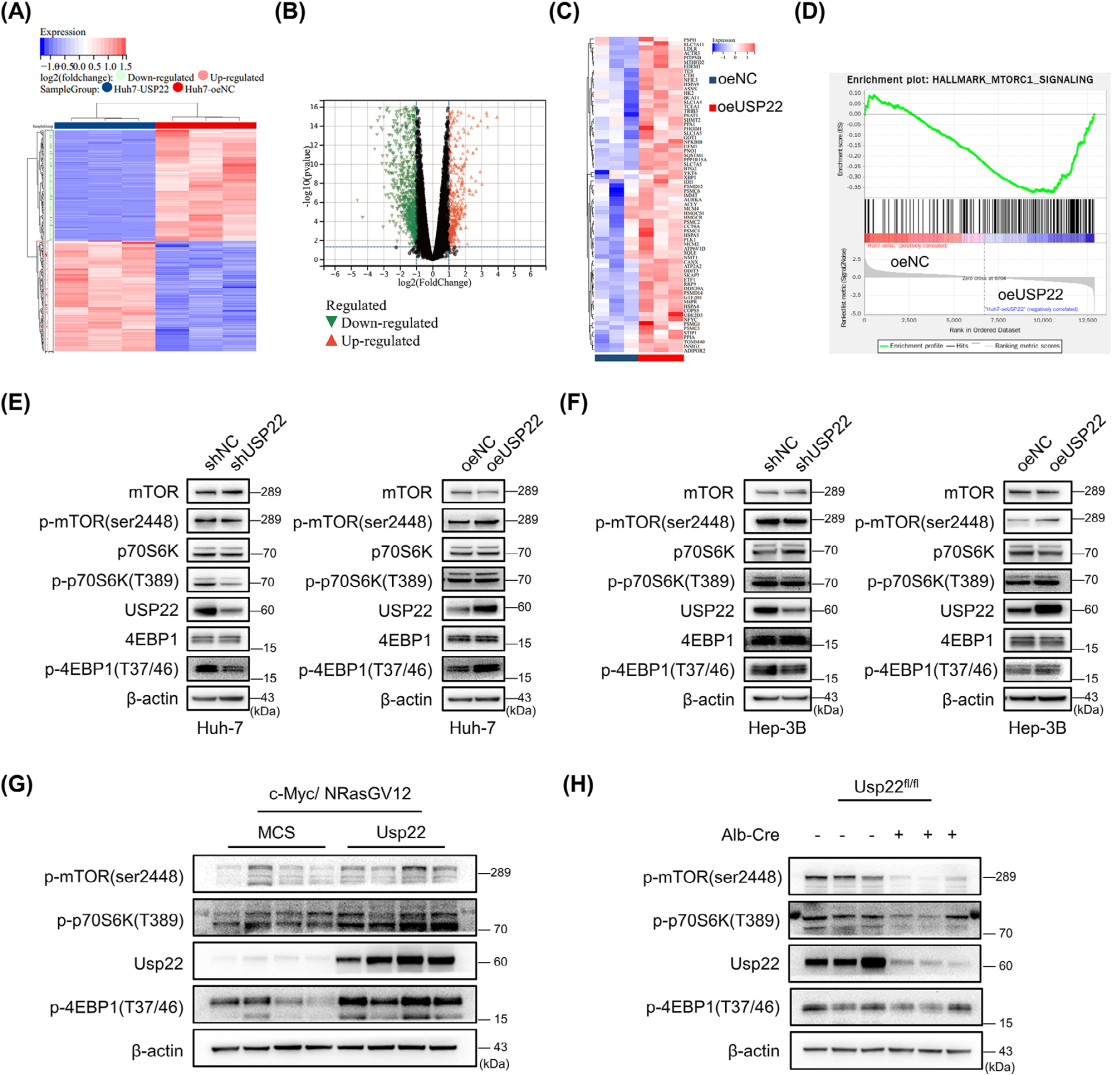
USP22 overexpression activates the mTORC1 pathway. (A) Heatmap of differentially expressed genes (DEGs) in Huh‐7 oeNC and oeUSP22 cells based on RNA sequencing data. (B) Volcano plots of DEGs in two cell lines. (C) The changes in key molecules in the mTORC1 pathway following USP22 overexpression are shown in the heatmap. (D) Gene set enrichment analysis (GSEA) revealed the activated mTORC1 pathway in the Huh‐7 oeUSP22 cells compared to oeNC cells (|normalized enrichment score| ≥ 1, *p* < 0.05, false discovery rate < 0.25). (E) Western blot results showing the key proteins of the mTORC1 pathway in Huh‐7 cells. (F) Western blot results showing the key proteins of the mTORC1 pathway in Hep‐3B cells. (G) Western blot results showing protein expression in c‐Myc/NrasGV12‐induced hepatocellular carcinoma tissues of mice with or without Usp22 overexpression. (H) Western blot results showing protein expressions in c‐Myc/NrasGV12‐induced tumor tissues of Usp22^fl/fl^; Alb‐Cre or Usp22^fl/fl^ mice. β‐Actin was used as a loading control.

To explore the downstream pathway, RNA sequencing was performed using human Huh‐7 oeNC and Huh‐7 oeUSP22 cell lines. Heatmaps and volcano plots of differentially expressed genes in the two cell lines are presented in Figure 2A,B. Gene set enrichment analysis (GSEA) revealed that the overexpression of USP22 increased mammalian target of rapamycin complex 1 (mTORC1) activity (Figure 2C,D). Furthermore, we explored critical phosphorylated proteins in the mTORC1 pathway using four HCC cell lines (Figures 2E,F and S2) and c‐Myc/NRasGV12 mouse HCC tissues (Figure 2G,H). The levels of these key phosphorylated proteins in the mTORC1 pathway were increased by USP22 overexpression and decreased upon its knockdown. Therefore, USP22 activates the mTORC1 signaling pathway both in vitro and in vivo.

### 2.3 USP22 promotes tumorigenic potential and sensitizes HCC toward rapamycin in vitro and in vivo

**FIGURE 3:**
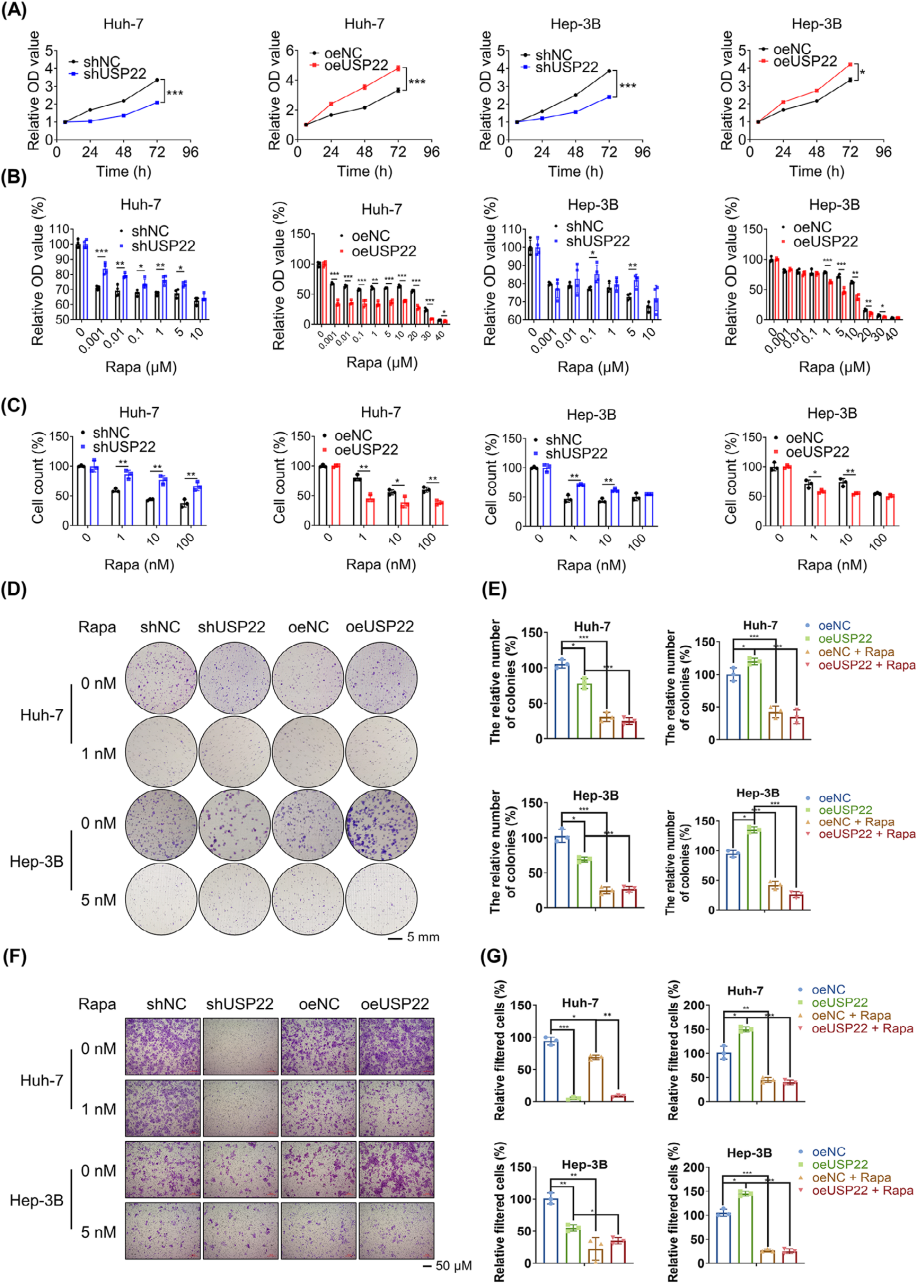
USP22 exerts tumor‐promoting functions and drives rapamycin sensitivity in hepatocellular carcinoma cells. (A) The effects of USP22 knockdown or overexpression on cell proliferation in Huh‐7 and Hep‐3B cells detected using a cell counting kit‐8 (CCK‐8) assay (*n* = 4). (B) The sensitivities of USP22 knockdown or overexpression cells toward rapamycin assessed in Huh‐7 and Hep‐3B cells using a CCK‐8 assay (*n* = 4). (C) Variations in rapamycin sensitivity detected by trypan blue staining‐based cell counts (*n* = 3). (D) Colony formation results and rapamycin sensitivity variations of Huh‐7 and Hep‐3B cells after USP22 knockdown or overexpression visualized by crystal violet staining. (E) The corresponding quantization diagrams of (D) (*n* = 3). (F) Migratory properties and variations in rapamycin sensitivity of Huh‐7 and Hep‐3B cells after USP22 knockdown or overexpression visualized by crystal violet staining. (G) The corresponding quantization diagrams of (F) (*n* = 3) (^*^ *p* < 0.05, ^**^ *p* < 0.01, ^***^ *p* < 0.001).

To investigate USP22 function in HCC cells, we performed gain‐ and loss‐of‐function studies. In the Huh‐7 and Hep‐3B cell lines, knockdown of USP22 slowed cell proliferation, while overexpression accelerated it (Figure 3A). The susceptibility of HCC cells to mTOR inhibitors was also investigated due to the altered mTORC1 pathway. Compared to control transfected cells (shNC or oeNC), treatment of HCC cells with different concentrations of rapamycin slightly affected the proliferation of USP22‐silenced (shUSP22) cells, but significantly reduced the proliferation of USP22 overexpression (oeUSP22) cells (Figure 3B). Consistent with the results of the cell counting kit‐8 (CCK‐8) assay, the cell counting data showed that oeUSP22 cells were more sensitive to rapamycin (Figure 3C). In addition, the colony formation abilities and migratory properties of the two HCC cell lines changed after silencing or overexpression of USP22 (Figure 3D–G), presenting the same trends as the CCK‐8 assays.

**FIGURE 4:**
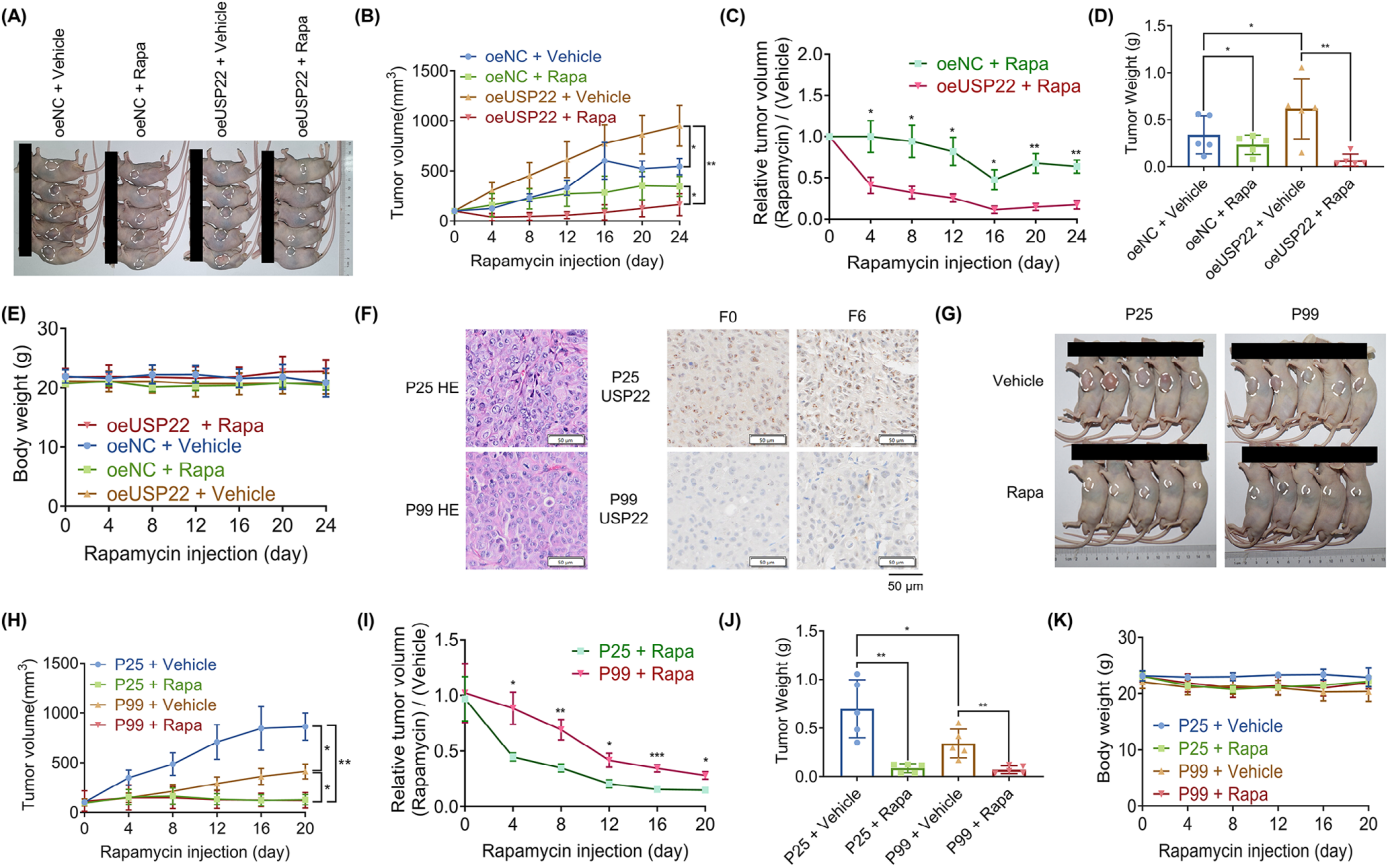
USP22 promotes tumor growth and sensitivity to rapamycin in vivo. (A) The tumor images of each group (*n* = 5). (B) Tumor volumes measured and compared every 4 days in each group. (C) The calculated relative tumor volume (Rapa/vehicle). Compared to the oeNC group, significant tumor regression was observed in the oeUSP22 group (*p* < 0.01). (D) Tumor weights of each group. (E) Mouse body weights of each group. There are no significant differences noted. (F) Hematoxylin–eosin staining and USP22 expression in two patient‐derived tumor xenograft models in the F0 and F6 generations. (G) Tumor images of each group (*n* = 5). (H) Tumor volumes of each group. (I) Comparison of the relative tumor volume (Rapa/vehicle). (J) Tumor weights of each group. (K) Mouse body weights of each group, there are no significant differences noted (^*^ *p* < 0.05, ^**^ *p* < 0.01, ^***^ *p* < 0.001).

Two xenograft mouse models were used to examine the role of USP22 in tumor growth in vivo. According to in vitro studies, Huh‐7 oeUSP22 cells promoted tumor development, and this effect was inhibited by rapamycin administration in mice (Figure 4A–E). Additionally, patient‐derived tumor xenograft (PDX) models with different expression levels of USP22 were used to evaluate the antitumor efficacy of rapamycin (Figure 4F). We found that PDX 25 with high USP22 expression exhibited larger tumor volumes and was more sensitive to rapamycin than PDX 99 with low USP22 expression (Figure 4G–K). These findings suggested that activation of the mTORC1 pathway after USP22 overexpression induces therapeutic susceptibility to rapamycin.

### 2.4 USP22 regulates FKBP12 to activate mTORC1 through deubiquitylation

**FIGURE 5:**
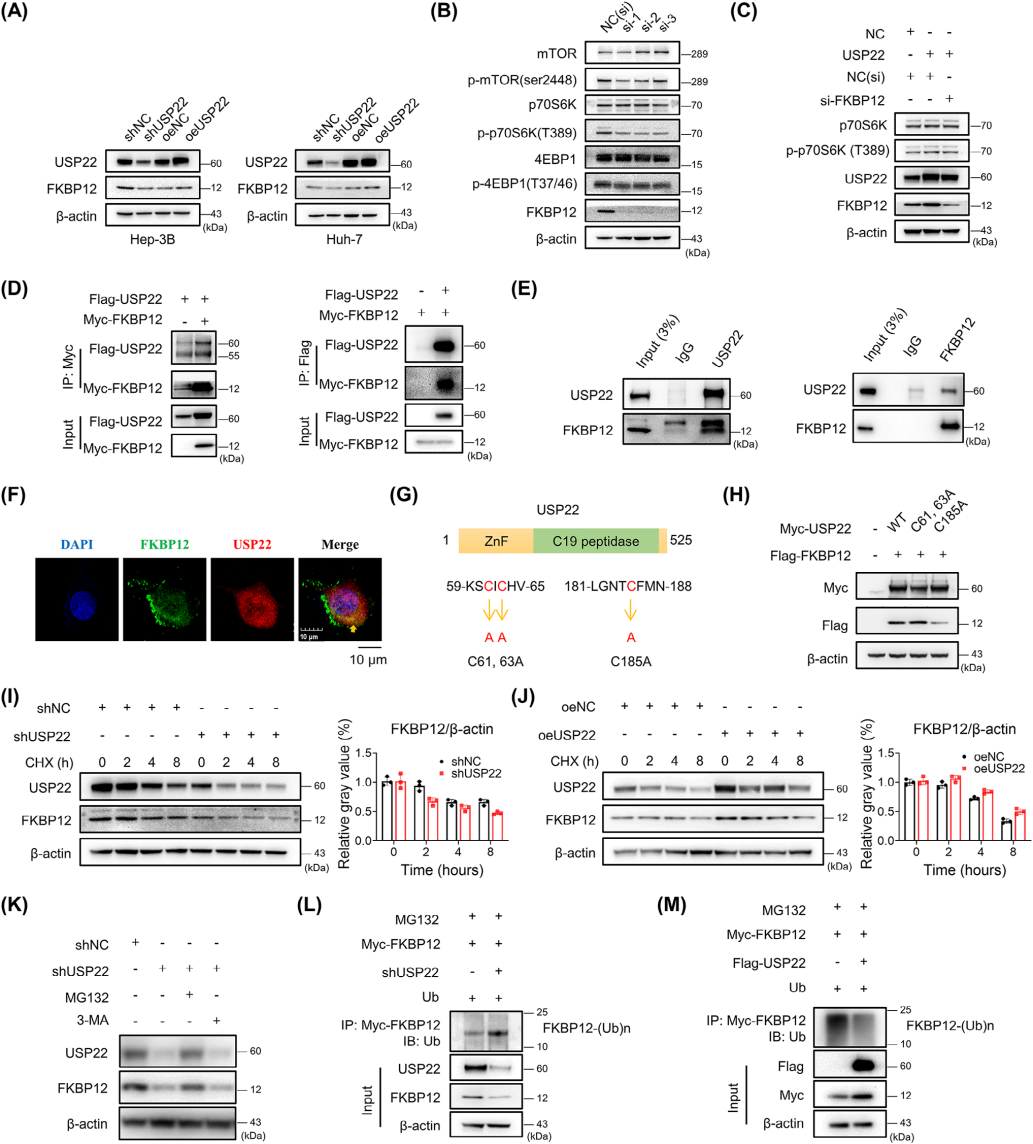
The effect of USP22 on FKBP12 protein stabilization and ubiquitination. (A) The change in FKBP12 protein in Hep‐3B and Huh‐7 cells. (B) Silencing of FKBP12 downregulated the proteins of the mTORC1 pathway in Hep‐3B cells. (C) Elevated p‐p70S6K (T389) in oeUSP22 cells could be rescued by additional silencing of FKBP12. (D) Isolated extracts from HEK293T cells obtained for immunoprecipitation (IP) using either an anti‐Myc or anti‐Flag antibody. (E) IP assay results of endogenous USP22 and FKBP12 in SNU‐449 cells. (F) Co‐localization of FKBP12 (green) with USP22 (red) observed using confocal microscopy and indicated by yellow arrow. (G) Illustration of USP22 and its point mutants. (H) HEK293T cells transfected with FKBP12‐Flag plasmids along with NC, Myc‐USP22 (wt), and each of the point mutants. (I) Western blot and ImageJ results in Hep‐3B shUSP22 or shNC cells after cycloheximide treatment (50 μg/mL). (J) Western blot and ImageJ results in SNU‐449 oeUSP22 or oeNC cells after cycloheximide treatment (50 μg/mL). (K) Western blot showing changes in FKBP12 after MG132 (10 μM, 2 h) or 3‐methyladenine (3‐MA) (5 mM, 12 h) treatment. (L) The ubiquitination of FKBP12 using IP and western blotting in Hep‐3B shUSP22 cells. (M) The ubiquitination of FKBP12 using IP and western blotting in HEK‐293T cells with USP22 overexpression.

Because USP22 was shown to activate mTORC1 activity, we hypothesized that USP22 interacts with certain proteins associated with mTORC1, including regulatory‐associated protein of mTOR (Raptor), mTOR‐associated protein, LST8 homolog (MLST8), mTOR, and FK506‐binding protein 12 (FKBP12). Immunoprecipitation (IP) was performed using an anti‐USP22 antibody to detect these proteins in SNU‐449 cells. The results showed that Raptor, MLST8, and mTOR did not interact with USP22 (Figure S3). Moreover, it is well‐established that FKBP12 inhibits mTORC1 activity by directly binding to rapamycin, and we found that USP22 overexpression induces therapeutic susceptibility to rapamycin. We examined FKBP12 and found that USP22 was an upstream regulator of FKBP12 (Figure 5A). Next, we assessed the effect of FKBP12 on the mTORC1 pathway. In Hep‐3B cells, the siRNA‐mediated knockdown of FKBP12 downregulated the effector proteins of the mTORC1 pathway (Figure 5B). Intriguingly, the activation of the mTORC1 pathway in oeUSP22 cells was rescued by the additional knockdown of FKBP12 (Figure 5C). These results indicated that the upregulation of USP22 activated the mTROC1 pathway via FKBP12.

Moreover, we identified an interaction between USP22 and FKBP12. Flag‐tagged USP22 and Myc‐tagged FKBP12 plasmids were used to transfect HEK293T cells. Co‐IP was performed in whole cell extracts with an anti‐Myc or anti‐Flag antibody (Figure 5D). The interaction between endogenous FKBP12 and USP22 in SNU‐449 cells is shown in Figure 5E, while the co‐localization of FKBP12 and USP22 was observed by confocal microscopy (Figure 5F). USP22 has a zinc finger domain at the N‐terminus and a ubiquitin‐specific peptidase domain at C19. We constructed USP22 point mutants (Figure 5G), and found that a point mutation in the ubiquitin‐specific peptidase domain of USP22 could not upregulate FKBP12 expression (Figure 5H). This finding implies that the ubiquitin‐specific peptidase domain is the key catalytic residue of USP22 involved in the regulation of FKBP12. USP22 is a deubiquitinating protease and might regulate FKBP12 function via deubiquitinase activity. Therefore, we explored the effect of USP22 on FKBP12 protein stability. USP22 silencing significantly facilitated FKBP12 protein degradation (Figure 5I), whereas USP22 overexpression delayed FKBP12 protein degradation (Figure 5J), suggesting that USP22 stabilized FKBP12. Besides, the proteasome inhibitor MG132, rather than the autophagy inhibitor 3‐methyladenine prevented the FKBP12 protein degradation in shUSP22 cells (Figure 5K), indicating that FKBP12 is degraded via the proteasomal pathway. In addition, knockdown of USP22 enhanced polyubiquitination‐induced FKBP12 degradation (Figure 5L), whereas USP22 overexpression reduced polyubiquitination‐induced FKBP12 degradation (Figure 5M). We further explored USP22 deubiquitination using a series of ubiquitin mutants (K6, K11, K27, K33, K48, and K63). The results indicated that the K63‐linked ubiquitination site alone did not influence FKBP12 polyubiquitination (Figure S4). In summary, USP22 activates mTORC1 by deubiquitinating FKBP12.

### 2.5 Activated mTORC1 further inhibits the autophagic degradation of USP22

**FIGURE 6:**
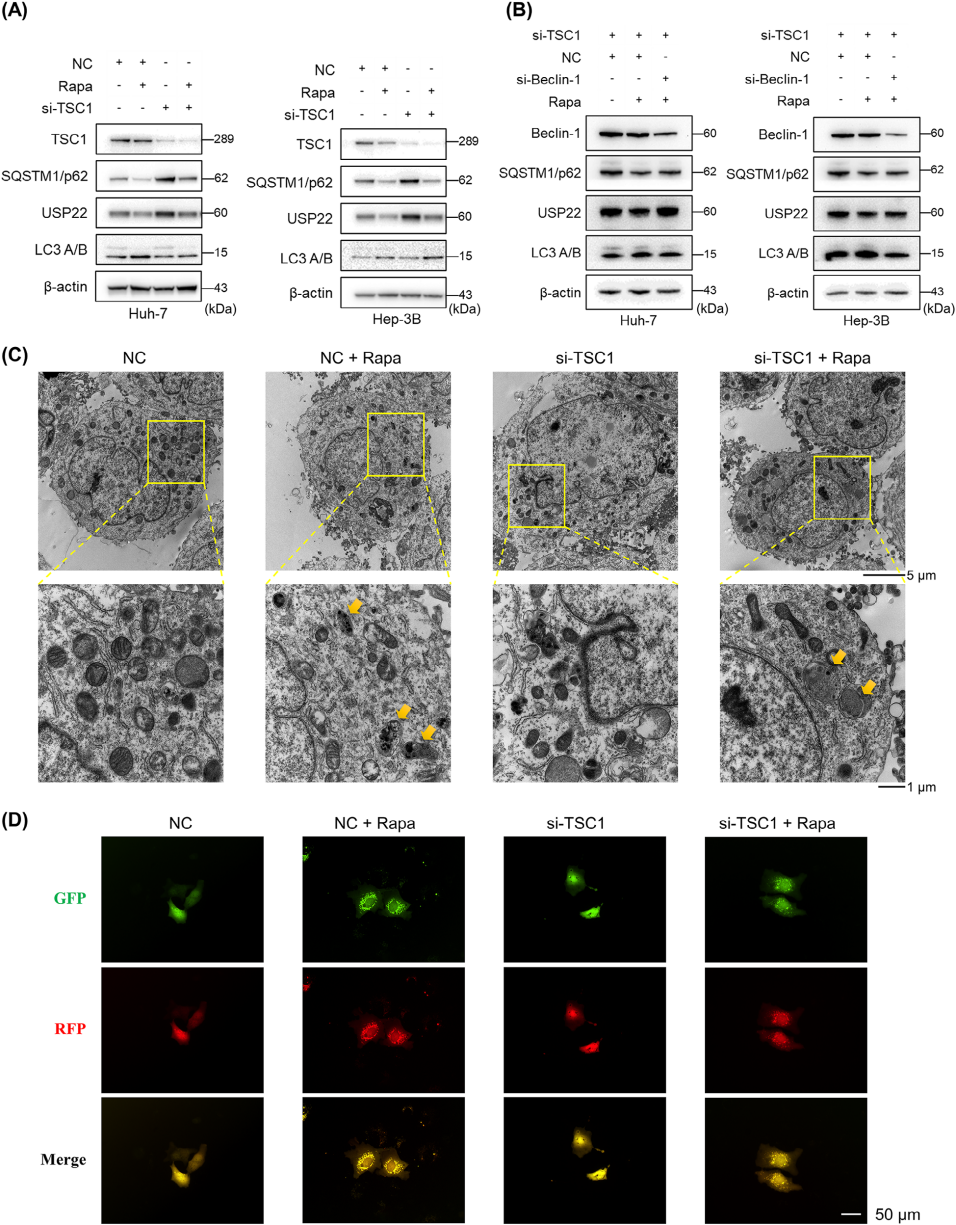
Activated mTORC1 stabilizes USP22 by inhibiting autophagic degradation. (A) Activated mTORC1 mediated by si‐tuberous sclerosis 1 (TSC1) stabilized USP22 protein level, and further rapamycin treatment diminished the upregulated USP22 and simultaneously activated autophagy in hepatocellular carcinoma cells. (B) Reduced protein level of USP22 with rapamycin treatment could be reversed by additional inhibition of autophagy (si‐Beclin‐1). (C) The autophagosome formations observed by transmission electron microscopy (yellow arrows). (D) Hep‐3B cells with or without knockdown of TSC1 transfected with mRFP‐GFP‐LC3 plasmid, and then treated with or without rapamycin. Yellow puncta represent autophagosomes and red puncta represent autolysosomes.

To further determine whether mTORC1 in turn regulates USP22, we inhibited it with rapamycin and found downregulated USP22 with enhanced autophagy activity (Figure 6A). In addition, activated mTORC1 mediated by the knockdown of tuberous sclerosis 1 (TSC1) stabilized USP22 and inhibited autophagy activity, and rapamycin treatment also reduced the upregulation of USP22 (Figure 6A). Importantly, the rapamycin‐induced decrease in USP22 expression was restored by blocking autophagy (Figure 6B). This indicates that activated mTORC1 stabilizes USP22 by inhibiting autophagic degradation. In addition, autophagosomes were observed by transmission electron microscopy (Figure 6C), and autophagic flux was detected by fluorescent LC3 in mRFP‐GFP‐LC3 transfected cells (Figure 6D). These results demonstrate that activated mTORC1 further stabilizes USP22 by inhibiting its autophagic degradation.

### 2.6 Clinical significance of USP22 in patients with HCC after LT

**FIGURE 7:**
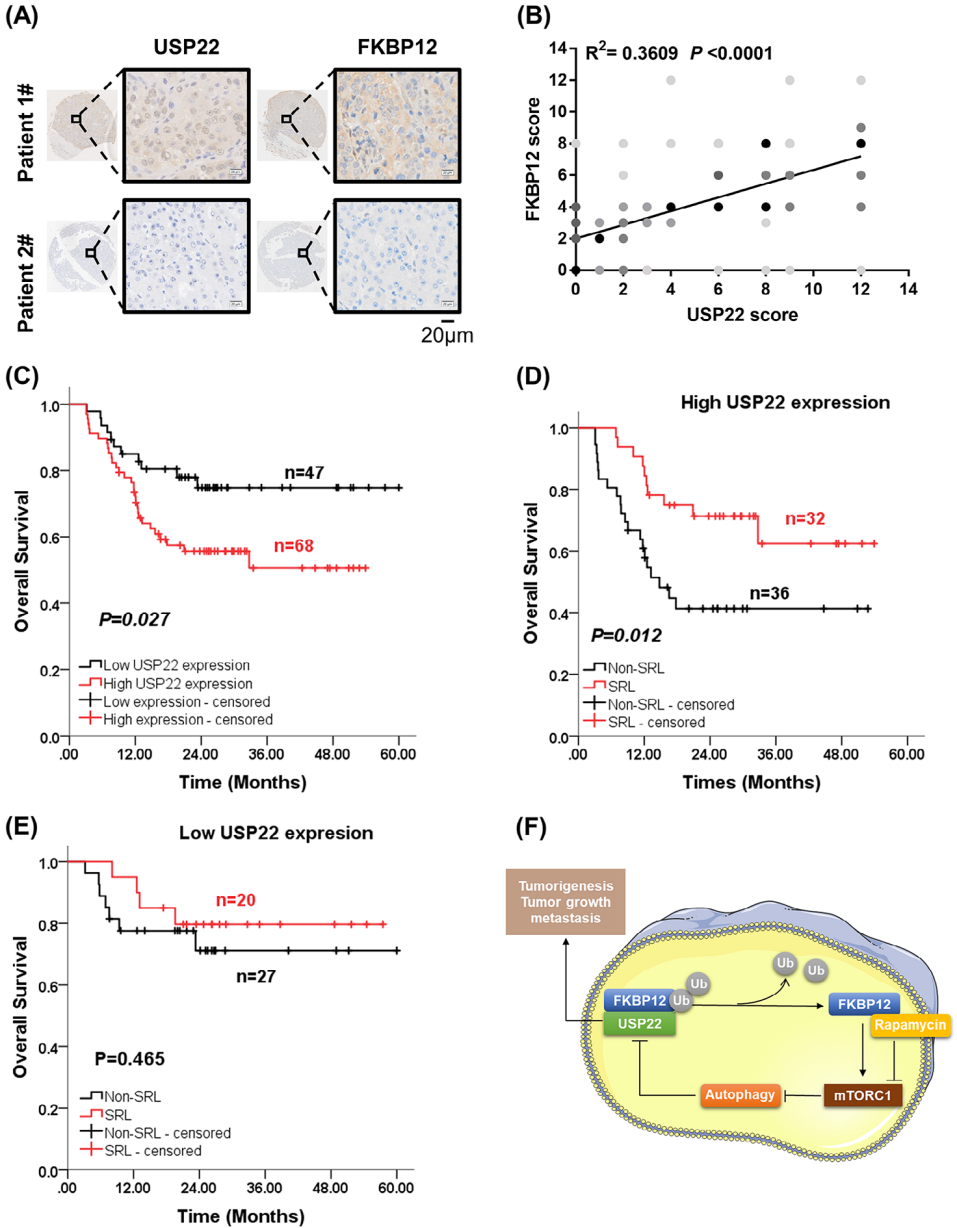
Clinical significance of USP22 in patients with hepatocellular carcinoma (HCC) after liver transplantation (LT). (A) Different expression levels of USP22 and FKBP12 detected using immunohistochemical staining in two LT recipients diagnosed with HCC. (B) Positive correlation between USP22 and FKBP12 expression in 205 HCC tissues (*R* ^2^ = 0.3609, *p* < 0.0001). (C) The effect of USP22 on the overall survival (OS) of LT recipients with HCC exceeding the Milan Criteria evaluated using the Kaplan–Meier method (*n* = 115, *p* = 0.027). (D) The effect of sirolimus (SRL) on the OS of recipients with high USP22 expression assessed using the Kaplan–Meier method (*n* = 68, *p* = 0.012). (E) The effect of SRL on the OS of recipients with low USP22 expression shown using the Kaplan–Meier method (*n* = 47, *p* = 0.465). (F) Diagram of the molecular mechanisms of USP22, FKBP12, mTORC1, and autophagy in HCC.

To explore the clinical significance of USP22, we detected the protein expression of USP22 and FKBP12 in 205 patients with HCC after LT using IHC. USP22 and FKBP12 were found to have a strong positive correlation (Figure 7A,B), further demonstrating their positive relationship.

Sirolimus is a common immunosuppressant administered after LT. Given that LT recipients fulfilling the Milan Criteria already had a satisfactory prognosis, we explored the role of USP22 in recipients beyond the Milan Criteria and especially investigated the effect of sirolimus on prognosis depending on USP22 expression. Table S1 presents the clinical features of the 115 LT recipients who did not meet the Milan Criteria, there were no notable variations in demographic factors. In LT recipients diagnosed with HCC beyond the Milan Criteria, those with high USP22 expression presented a lower 3‐year overall survival (OS) rate than those with low USP22 expression (74.8% vs. 50.6%, *p* = 0.027) (Figure 7C). Univariate and multivariate Cox regression analyses indicated that the use of sirolimus was an independent protective factor for OS (Hazard Ratio (HR) = 0.465, *p* = 0.035), whereas vascular invasion (HR = 2.513, *p* = 0.015) and Child–Pugh score (high) (HR = 1.327, *p* = 0.023) were independent risk factors (Table S2).

Further subgroup analysis was performed based on USP22 expression. The clinical features of these recipients are shown in Table S3. In recipients with high USP22 expression, sirolimus improved the 3‐year OS (62.4% vs. 41.3%, *p* = 0.012) (Figure 7D) and was an independent protective factor for OS (HR = 0.268, *p* = 0.005) (Table S4). However, sirolimus did not improve OS in recipients with low USP22 expression (Figure 7E). Therefore, high USP22 expression in tumor tissues could predict the benefit of sirolimus in patients with HCC after LT, which supports the significant role of USP22 in activating mTORC1 in HCC.

In summary, in HCC, USP22 overexpression increased mTORC1 activity and sensitized HCC toward rapamycin. Mechanistically, USP22 interacted with FKBP12 to activate the mTORC1 pathway through deubiquitylation. Increased mTORC1 activity further stabilized USP22 by inhibiting autophagic degradation (Figure 7F).

## 3 DISCUSSION

In the current study, we demonstrated that the ubiquitin hydrolase USP22 contributed to HCC tumorigenesis and promoted tumorigenic potential of HCC, suggesting an oncogenic role of USP22 consistent with the majority of published studies.^17^, ^18^, ^19^, ^20^ Moreover, overexpression of USP22 alone was not sufficient to drive tumor formation in mice but disrupted several cancer‐related pathways.^21^ USP22 overexpression accelerated c‐Myc/NRasGV12‐induced HCC in this study. To the best of our knowledge, this is the first study to provide direct evidence of an association between USP22 and tumorigenesis in orthotopic HCC. This is an essential continuation of our previous study.^13^ USP22 has also been reported to have antitumor effects.^14^, ^15^ The dual functions of USP22 in tumors may be attributed to its tissue specificity and unique cellular environment, thereby underscoring the intricate nature of its underlying mechanism.

Somatic mutations in TP53 are among the most frequent alterations in human malignancies. In a Chinese cohort of patients with hepatitis B virus‐related HCC, the mutation frequency of TP53 was 58%.^22^ Additionally, as a key tumor suppressor gene, TP53 haploinsufficiency was reported to facilitate mTOR signaling and promote HCC tumorigenesis.^23^ In the absence of TP53, we found that USP22 enhanced hypoxia‐induced stemness,^13^ which facilitated us to further investigate the relationship between USP22 and hepatotumorigenesis. However, this study did not explore the role of USP22 in HCC tumorigenesis in the context of TP53 mutations. Relevant studies in this area are currently under way.

It is crucial to elucidate the mechanisms underlying the oncogenic role of USP22 in HCC. One of our central findings was the activation of the mTORC1 pathway following USP22 overexpression. Indeed, we demonstrated that USP22 overexpression sensitized HCC cells and xenograft models to rapamycin treatment. Moreover, USP22 was found to interact with FKBP12, a 12 kDa enzyme that was initially reported to bind to the immunosuppressors FK506 and rapamycin. It was well established that FKBP12 inhibited mTORC1 activity by directly binding to the FKBP12‐rapamycin binding domain with rapamycin.^24^ In addition, FKBP12 has many other functions that involve binding to different cellular receptors or targets.^25^, ^26^, ^27^ Hu et al. reported that mTOR was more active in FKBP12‐depleted cells in both the presence and absence of rapamycin.^28^ However, in this study, we found that the reduction in FKBP12 decreased the protein levels of the mTORC1 pathway. We attribute this result to the potential context‐dependent function of FKBP12. The expression of FKBP12 was decreased in breast cancer tissues, and low FKBP12 expression was associated with poor prognosis.^29^ In contrast, it was upregulated in HCC tissues, and high FKBP12 expression predicted poor OS according to The Cancer Genome Atlas data and our findings (data not shown). These results suggested that the role of FKBP12 in cancer was not as black and white as previously assumed, and its relative level might be critical for mTORC1 inhibition.^30^ Thus, inhibition of the mTORC1 pathway mediated by FKBP12 knockdown was reasonable in HCC cells. FKBP12 is a novel downstream substrate of USP22. The upregulation of FKBP12 partially explains the interaction between USP22 and mTORC1, especially in HCC with high USP22 expression, which exhibits heightened sensitivity to rapamycin.

Autophagy acts as a scavenger and is one of the important ways of protein degradation in cells.^31^ Autophagy plays a dual role in HCC carcinogenesis. It inhibits the initiation of carcinogenesis while promoting tumor growth and metastasis during tumor progression.^32^, ^33^ To the best of our knowledge, only a few studies have investigated the regulatory mechanisms underlying USP22 expression and activity, particularly at the protein level.^3^ In this study, we found for the first time that activation of autophagy led to the degradation of USP22 and that activated mTORC1 stabilized USP22 by inhibiting autophagic degradation. Therefore, autophagy may inhibit tumor initiation by downregulating USP22 expression in HCC.

The survival benefits of mTOR inhibitors have been proven in LT recipients with HCC. The outcomes of the only phase 3, multicenter, randomized trial SiLVER disappointed clinicians,^34^ but the reanalysis results revealed that in recipients with higher tumor activity or treatment with sirolimus ≥3 months improved prognosis in LT for HCC.^35^ Our previous study yielded similar results. We found that LT recipients with HCC exceeding the Milan Criteria could benefit from sirolimus‐based immunosuppression,^36^, ^37^ and the low TSC1/2 expression subgroup (higher tumor activity) benefited the most.^38^, ^39^ Therefore, USP22 expression levels could help stratify recipients, who might benefit from sirolimus. Here, we presented that sirolimus‐based immunosuppression could improve OS in LT recipients beyond the Milan Criteria with high USP22 expression, which was in accordance with in vitro and in vivo experiments. Moreover, USP22/mTORC1 formed a positive feedback loop, which well explained the superior therapeutic efficacy of sirolimus in the USP22 overexpression subgroup of patients. Therefore, mTORC1 inhibition might be a potentially effective therapeutic strategy for patients with HCC with high USP22 expression. Although this retrospective study has same limitations (such as small sample size, and selection bias), we believe that it would be helpful for selecting eligible recipients for anti‐mTOR‐based therapies after LT.

In summary, USP22 exerted a protumorigenic role by activating mTORC1 and sensitizing HCC to rapamycin. Mechanistically, USP22 interacted with FKBP12 and stabilized it via deubiquitylation. Furthermore, activated mTORC1 in turn upregulated USP22 by inhibiting autophagic degradation. Clinically, high USP22 expression predicted poor prognosis for LT recipients with HCC beyond the Milan Criteria, and those patients could benefit the most from sirolimus, indicating that USP22 might serve as a proper stratification factor to give some advice for the clinical use of sirolimus in post‐LT management.

## 4 CONCLUSIONS

Taken together, USP22 accelerates c‐Myc/NrasGV12‐induced tumorigenesis and promotes tumor progression through an FKBP12/mTORC1/autophagy positive feedback loop in HCC cells. FKBP12 is a newly identified substrate of USP22, and the activation of autophagy to degrade the USP22 protein is reported here for the first time. Clinically, USP22 may serve as an effective biomarker for selecting eligible recipients with HCC for anti‐mTOR‐based therapy after LT.

## 5 MATERIALS AND METHODS

### 5.1 Generation of mice and genotyping

Usp22 conditional knockout mice were purchased from Cyagen Biosciences. To produce targeted conditional knockout offspring, the gRNA for Usp22 gene in mice (Table S5), along with the donor vector containing loxP sites, and Cas9 mRNA, were simultaneously injected into fertilized mouse eggs. F0 founder animals were identified using polymerase chain reaction followed by sequence analysis, and then bred with wild‐type mice to assess germline transmission for F1 animal generation. To generate F2, an F1 targeted mouse was crossed with a tissue‐specific Alb‐Cre deletion mouse. Heterozygous Cre^+^ mice were then bred with homozygous mice. Genotyping was performed using the Mouse Tail Quick Genotyping Kit (Beyotime) with appropriate primers (Table S6) and some results are shown in Figure S5.

### 5.2 Plasmid and lentiviral construction

The plasmids (RiboBio) used in animal and cell experiments are listed in Table S7. All plasmids were purified using an Endo‐Free Plasmid Max Kit (Omega Bio‐Tek). Lentivirus overexpressing USP22 and shRNAs against USP22 were provided by Shandong Vigene. Four separate siRNAs targeting USP22 were created, validated, and effectively packaged in a four in one shUSP22 lentivirus to prevent off‐target effects (Figure S6). All siRNA sequences are listed in Table S8.

### 5.3 Hydrodynamic tail vein injection models

C57BL/6J mice (6−8 weeks, male) were obtained from the Shanghai Experimental Animal Centre, Chinese Academy of Science, and Usp22^fl/fl^; Alb‐Cre mice were purchased from Cyagen Biosciences. Briefly, mice were administered a hydrodynamic tail vein injection of plasmid mixture in 2 mL saline within 5–9 s as previously described.^40^ Each plasmid mixture contained the following: c‐Myc 10 μg, NRasGV12 10 μg, SB100 0.8 μg, MCS 20 μg, and Flag‐USP22 20 μg. Body weight, abdominal palpation, and physiological parameters of the mice were recorded daily. Mice were euthanized when a high burden of HCC manifested, for example, an abdominal mass.

### 5.4 Immunohistochemical analysis

Tumors from an orthotopic HCC mouse model were isolated, treated with formalin, and embedded in paraffin. A total of 205 HCC samples were obtained to establish a microarray. The manufacturer's instructions for the Immunohistochemical Assay Kit (Abcam) were followed during immunohistochemical staining. The antibodies used are listed in Table S9. An Olympus VS200 was used to scan the microarray slides and they were assessed using the immunoreactive score (IRS), which was determined by multiplying the staining intensity grades and the fraction of positive cells. The gradations were ranked as shown in Table S10. IRS (0−7) and IRS (8−12) were analyzed for low and high expression levels, respectively.

### 5.5 Cell culture, transfection, and lentivirus infection

Human HCC cell lines (Huh‐7, Hep‐G2, and Hep‐3B) and HEK293T cells were acquired from the Shanghai Institute of Cell Biology, Chinese Academy of Sciences, and SNU‐449 cells were purchased from Guangzhou Cellbook Biotech. The authenticity of all cells was verified using short tandem repeat profiling. Huh‐7 and HEK293T cells were grown in Dulbecco's modified Eagle's medium with high glucose (BI), Hep‐G2 and Hep‐3B in minimum essential medium, and SNU‐449 in RPMI‐1640. Fetal bovine serum (FBS, 10%; Gibco), 100 U/mL penicillin, 100 μg/mL streptomycin, and 50 μg/mL gentamicin (Solarbio) were added to the media. All cells were cultured in a 37°C, 5% CO_2_ incubator. The manufacturer's protocol (Polyplus) was followed for transient siRNA and DNA transfection using jetPRIME. Lentivirus infection was performed as previously reported.^41^

### 5.6 RNA sequencing and GSEA

The poly (A) selection procedure was used to select mRNA from the HCC cell line Huh‐7 after extracting total RNA. RiboBio prepared and sequenced the RNA sequencing library.^42^ DESeq2 was used for differential expression analysis, employing a significance threshold of *p* < 0.05 and a minimum fold change of 1.5. Cluster analysis was conducted to determine the expression patterns of various genes under diverse experimental conditions.

### 5.7 Cell viability assay

Cell viability was determined using a CCK‐8 assay. Briefly, 2000−5000 HCC cells were seeded in 96‐well plates, cultured for 6, 24, 48, and 72 h with or without rapamycin, and treated with CCK‐8 solution. After incubation at 37°C with 5% CO_2_ for 1–2 h, the absorbance was measured at 450 nm with an EnSpire 2300 Multilabel Reader (PerkinElmer). For cell counting, 2−5 × 10^4^ cells were seeded into 12‐well plates. Following a 48‐h treatment period, with or without rapamycin, the cells were digested, resuspended, and subsequently stained with trypan blue. The cells were counted using a cell‐counting plate under a microscope.

### 5.8 Colony formation assay

Two thousand HCC cells were seeded in 24‐well plates and treated with various concentrations of rapamycin for 10−14 days. The cell colonies were fixed with methanol and visualized using crystal violet staining.

### 5.9 Cell migration assay

A total of 50,000 HCC cells were diluted in 200 μL of media without serum and containing 0.1% bovine serum albumin (BSA). These cells, with or without rapamycin, were introduced into the upper chamber of a transwell plate (Corning). The lower compartment received 700 μL of media containing 10% FBS, either with or without rapamycin. After 24–48 h, the polycarbonate membranes were washed, fixed, and stained with 0.1% crystal violet. The cells were then examined under a microscope. The experiments were independently repeated three times.

### 5.10 Western blotting

Total protein was extracted from the cells or tissues using RIPA lysis buffer (FUDE) supplemented with protease and phosphatase inhibitor cocktails (Roche). Electrophoresis was performed on 4%−20% sodium dodecyl–sulfate polyacrylamide gel electrophoresis gels (GenScript). The proteins were then transferred to polyvinylidene difluoride membranes (Millipore). Following gentle shaking in blocking buffer (Tris‐buffered saline with 5% nonfat milk or BSA) for 1 h, the blots were incubated with primary antibody at 4°C overnight and subsequently with secondary antibody at 37°C for 1 h. The antibodies used are listed in Table S9. The bands were visualized using ECL chemiluminescence (FUDE) and a Fluor Chem M imaging system (ProteinSimple).

### 5.11 Animal experiments

For the cell‐derived xenograft model, 5 × 10^6^ Huh‐7 oeNC and Huh‐7 oeUSP22 cells were subcutaneously implanted into 5‐week‐old male BALB/c nude mice. Details of the observations, rapamycin solubilization, and data analyses were performed as previously described.^38^ Rapamycin was administered daily via intraperitoneal injection at a dose of 1 mg/kg. For the PDX model, fresh HCC tissues were acquired from patients, diced into 1‐mm^3^ pieces and implanted into the flanks of 5‐week‐old male NOD‐SCID mice purchased from the Shanghai Experimental Animal Centre, Chinese Academy of Science. Mice that successfully developed subcutaneous tumors from the tumors of the original patients were defined as founder 0 (F0). The second generation of PDX model was defined as F1, and so forth. PDX from patient 25 (PDX 25) and patient 99 (PDX 99) had different expression levels of USP22 and were chosen for the assessment of the antitumor effect of rapamycin.

### 5.12 Co‐IP assay

In brief, cell lysates were precleaned using IgG and Protein A+G agarose (Beyotime) for 30 min and then immunoprecipitated with proper antibodies against USP22, FBKP12, Flag, or Myc (Table S9) by incubation with agarose at 4°C for 2 h. Next, the beads were washed three times with the lysis buffer. Precipitated proteins (10 μg) were prepared for western blot analysis, and the procedure was as described above.

### 5.13 Immunofluorescence

Glass coverslips were attached to six‐well plates and the cells were placed on them. Fluorochrome‐conjugated secondary antibodies were used to identify target proteins. Nuclear localization was achieved using DAPI (300 nM) labeling. Fluorescence distribution was observed using a laser scanning confocal microscope (Olympus).

### 5.14 Ubiquitination assay

To explore ubiquitination, the protein synthesis inhibitor cycloheximide and the proteasome inhibitor MG132 (MCE) were used. Cells were disrupted in a solution containing 50 mM Tris (pH 7.4), 150 mM NaCl, 1% NP‐40, 0.5% sodium deoxycholate, and 0.1% sodium dodecyl sulfate along with cocktails. The samples were heated at 95°C for 5 min to cause denaturation. IP and western blotting were performed as previously described. The antibodies used are shown in Table S9.

### 5.15 Patients and data sources

In total, 205 adult patients with HCC who received deceased donor LT at the First Affiliated Hospital of Zhejiang University between January 1, 2015, and December 30, 2022, were enrolled in this part of the study. Data from the China Liver Transplant Registry database (http://www.cltr.org) were collected for further analyses. IHC and IRS, as described above, were used to analyze the expression of USP22 and FKBP12. A total of 115 recipients beyond the Milan Criteria were further divided into high/low USP22 expression and sirolimus (SRL)/non‐SRL groups. Patients with pathologically diagnosed HCC and complete follow‐up records were included in this study. Exclusion criteria included the use of SRL for less than 30 days or after HCC recurrence, death within 90 days, and multiple‐organ transplantation.

### 5.16 Immunosuppression

The immunosuppressants were the same as detailed previously.^38^ After LT, tacrolimus/cyclosporine A (CNI) was administered to the non‐SRL group, and dose adjustments were made based on blood concentration and liver function. Following surgery, the target level of tacrolimus in the peripheral blood was 4–8 ng/mL. Additionally, clinicians lowered the tacrolimus dose as much as feasible, as long as liver function was normal. In the SRL group, CNI was administered initially, then 30−45 days after LT, SRL was administered. When SRL was introduced, the dose of CNI was halved and stopped when SRL reached the desired blood concentration (4–10 ng/mL). Both groups continued to receive mycophenolate.

### 5.17 Statistical analysis

IBM SPSS Statistics 23.0 and GraphPad Prism 7.00 were used for the statistical analysis. Data are displayed as mean ± standard deviation. Student's *t*‐test and the Mann–Whitney *U*‐test were used for comparisons between two groups, and Pearson's chi‐square test was applied for categorical variables. The Kaplan–Meier method was used to compare OS, while differences between groups were evaluated using a log‐rank test. Cox univariate analysis included all factors with *p*‐values less than 0.05, which were then incorporated into multivariate analysis using the Cox proportional hazards model. Significance was defined as *p* < 0.05 for all two‐tailed *p*‐values.

## AUTHOR CONTRIBUTIONS

S.Z., S.L., and X.X. conceived and supervised the study. Q.Y., W.Z., and S.X. performed the experiments. Q.Q., Q.Z., and L.Z. analyzed and interpreted the data. Q.Y. and W.Z., with the help of S.Z., S.L., and X.X., wrote and revised the manuscript. All the authors have read and approved the final manuscript.

## CONFLICT OF INTEREST STATEMENT

The authors declare they have no conflicts of interest.

## ETHICS STATEMENT

In PDX models, written informed consent was obtained from all participants. The animal experiments were approved by the Ethics Committee of Zhejiang University School of Medicine, strictly in accordance with the National Institute Guide for the Care and Use of Laboratory Animals (approval number: ZJU20230324). The study involving human data and tissue was approved by the Ethics Committee of the First Affiliated Hospital, Zhejiang University School of Medicine, strictly under the guidelines of the 2013 Declaration of Helsinki (approval number: 2018768).

## Supporting information

Supporting Information

## Data Availability

The datasets used and/or analyzed during the current study are available from the corresponding author upon reasonable request.
