# Supplementary material for: Ubiquitin‐specific protease 22 promotes tumorigenesis and progression by an FKBP12/mTORC1/autophagy positive feedback loop in hepatocellular carcinoma
Source: MedComm (2020). 2023 Dec 1;4(6):e439. doi: 10.1002/mco2.439 (PMC10691294; doi:10.1002/mco2.439)
Supplement: Supplementary file 1 — Supporting Information [file MCO2-4-e439-s001.docx]

**Title:**

Ubiquitin-specific protease 22 promotes tumorigenesis and progression by an FKBP12/mTORC1/autophagy positive feedback loop in hepatocellular carcinoma

**Running Title:** USP22 promotes tumorigenesis and progression

**Authors and Afﬁliations:**

Qianwei Ye^1,2,3,4^^#^, Wei Zhou^2,3#^, Shengjun Xu^1,3#^, Qingyang Que^2,3^, Qifan Zhan^2,3^, Lincheng Zhang^2,3^, Shusen Zheng^4,5^, Sunbin Ling^1,2,3*^, Xiao Xu^2,3*^

1. Department of General Surgery, Hangzhou First People’s Hospital, Hangzhou, 310006, China.
2. Zhejiang University School of Medicine, Hangzhou 310058, China.
3. Key Laboratory of Integrated Oncology and Intelligent Medicine of Zhejiang Province, Hangzhou, 310006, China.
4. NHC Key Laboratory of Combined Multi-organ Transplantation, Hangzhou, 310003, China.
5. State Key Laboratory for Diagnosis and Treatment of Infectious Diseases, The First Affiliated Hospital, School of Medicine, Zhejiang University, Hangzhou, 310003, China.

#These authors contributed equally to this work

**Correspondence to**

Xiao Xu, MD, Zhejiang University School of Medicine, Hangzhou 310058, China. Email: zjxu@zju.edu.cn;

And Sunbin Ling, MD, Department of General Surgery, Hangzhou First People’s Hospital, Hangzhou, 310006, China. Email: lsb0330@zju.edu.cn.

Table S1 Characteristics of LT recipients with HCC beyond the Milan criteria based on USP22 expression

| Variable | All (n=115) | Low USP22 expression (n=47) | High USP22 expression(n=68) | *P* value |
| --- | --- | --- | --- | --- |
| Age |  |  |  | 0.335 |
| ≥ 50 years old | 69 (60%) | 31 (66%) | 38 (55.9%) |  |
| < 50 years old | 46 (40%) | 16 (34%) | 30 (44.1%) |  |
| Gender |  |  |  | 0.066 |
| Males | 112 (97.4%) | 44 (93.6%) | 68 (100%) |  |
| Females | 3 (2.6%) | 3 (6.4%) | 0 (0%) |  |
| Pathological grade |  |  |  | 1.000 |
| High or Mod | 58 (50.4%) | 24 (51.1%) | 34 (50.0%) |  |
| Low | 57 (57.0%) | 23 (48.9%) | 34 (50.0%) |  |
| Tumor nodes |  |  |  | 0.803 |
| 1 | 20 (17.4%) | 9 (19.1%) | 11 (16.2%) |  |
| ≥ 2 | 95 (82.6%) | 38 (80.9%) | 57 (83.8%) |  |
| Vascular invasion |  |  |  | 0.569 |
| Yes | 61 (53.0%) | 23 (48.9%) | 38 (55.9%) |  |
| No | 54 (47.0%) | 24 (51.1%) | 30 (44.1%) |  |
| Largest tumor size |  |  |  | 0.524 |
| ≥ 5cm | 105 (91.3%) | 44 (93.6%) | 61 (89.7%) |  |
| < 5cm | 10 (8.7%) | 3 (6.4%) | 7 (10.3%) |  |
| AFP |  |  |  | 1.000 |
| ≥ 400 ng/mL | 39 (33.9%) | 16 (34.0%) | 23 (33.8%) |  |
| < 400 ng/mL | 76 (66.1%) | 31 (66.0%) | 45 (66.2%) |  |
| TACE before LT |  |  |  | 1.000 |
| Yes | 47 (40.9%) | 19 (40.4%) | 28 (41.2%) |  |
| No | 68 (59.1%) | 28 (59.6%) | 40 (58.8%) |  |
| RFA before LT |  |  |  | 0.738 |
| Yes | 10 (8.7%) | 5 (10.6%) | 5 (7.4%) |  |
| No | 105 (91.3%) | 42 (89.4%) | 63 (92.6%) |  |
| MELD | 20.51±11.14 | 19.13±11.13 | 21.47±11.14 | 0.289 |
| Child-Pugh | 8.66±2.14 | 8.49±2.00 | 8.78±2.23 | 0.277 |
| Capsule |  |  |  | 1.000 |
| Complete | 61 (53.0%) | 25 (53.2%) | 36 (52.9%) |  |
| Incomplete | 54 (47.0%) | 22 (46.8%) | 32 (47.1%) |  |
| HBV |  |  |  | 0.777 |
| + | 101 (87.8%) | 42 (89.4%) | 59 (86.8%) |  |
| - | 14 (12.2%) | 5 (10.6%) | 9 (13.2%) |  |

Table S2 Univariate and multivariate Cox regression analysis of risk factors for OS in recipients beyond the Milan criteria

| Variable | Univariate analysis | | |  | Multivariate analysis | | | |
| --- | --- | --- | --- | --- | --- | --- | --- | --- |
|  | Exp(B) | 95% CI | *P* | |  | Exp (B) | 95% CI | *P* |
| Use of sirolimus | 0.469 | 0.245-0.896 | ***0.022*** | |  | 0.465 | 0.228-0.949 | ***0.035*** |
| Age | 1.023 | 0.546-1.916 | 0.944 | |  |  |  |  |
| Gender (female) | 0.727 | 0.100-5.290 | 0.753 | |  |  |  |  |
| Pathological grade (low) | 2.543 | 1.316-4.913 | ***0.005*** | |  |  |  |  |
| Tumor nodes ≥ 2 | 2.205 | 0.786-6.188 | 0.133 | |  |  |  |  |
| Vascular invasion | 2.670 | 1.361-5.239 | ***0.004*** | |  | 2.513 | 1.199-5.266 | ***0.015*** |
| Largest tumor size≥ 5cm | 4.473 | 0.615-32.544 | 0.139 | |  |  |  |  |
| AFP ≥ 400 ng/mL | 2.869 | 1.548-5.318 | ***0.001*** | |  |  |  |  |
| TACE before LT | 1.025 | 0.552-1.903 | 0.938 | |  |  |  |  |
| RFA before LT | 0.720 | 0.222-2.334 | 0.585 | |  |  |  |  |
| MELD | 1.038 | 1.011-1.066 | ***0.005*** | |  |  |  |  |
| Child-Pugh | 1.289 | 1.104-1.505 | ***0.001*** | |  | 1.327 | 1.040-1.693 | ***0.023*** |
| Capsule (Complete) | 0.577 | 0.307-1.082 | 0.086 | |  |  |  |  |
| HBV | 2.056 | 0.634-6.670 | 0.230 | |  |  |  |  |

Table S3 Characteristics of recipients with high USP22 expression

| Variable | All (n=68) | Non-SRL group  (n=36) | SRL group  (n=32) | *P* value |
| --- | --- | --- | --- | --- |
| Age |  |  |  | 0.222 |
| ≥ 50 years old | 38 (55.9%) | 23 (63.9%) | 15 (46.9%) |  |
| < 50 years old | 30 (44.1%) | 13 (36.1%) | 17 (53.1%) |  |
| Gender |  |  |  | / |
| Males | 68 (100%) | 36 (100%) | 32 (100%) |  |
| Females | 0 (0%) | 0 (0%) | 0 (0%) |  |
| Pathological grade |  |  |  | 0.808 |
| High or Mod | 34 (50.0%) | 19 (52.8%) | 15 (46.9%) |  |
| Low | 34 (50.0%) | 17 (47.2%) | 17 (53.1%) |  |
| Tumor nodes |  |  |  | 0.744 |
| 1 | 11 (16.2%) | 5 (13.9%) | 6 (18.8%) |  |
| ≥ 2 | 57 (83.8%) | 31 (86.1%) | 26 (81.3%) |  |
| Vascular invasion |  |  |  | 0.807 |
| Yes | 38 (55.9%) | 21 (58.3%) | 17 (53.1%) |  |
| No | 30 (44.1%) | 15 (41.7%) | 15 (46.9%) |  |
| Largest tumor size |  |  |  | 0.241 |
| ≥ 5cm | 61 (89.7%) | 34 (94.4%) | 27 (84.4%) |  |
| < 5cm | 7 (10.3%) | 2 (5.6%) | 5 (15.6%) |  |
| AFP |  |  |  | 0.444 |
| ≥ 400 ng/mL | 23 (33.8%) | 14 (38.9%) | 9 (28.1%) |  |
| < 400 ng/mL | 45 (66.2%) | 22 (61.1%) | 23 (71.9%) |  |
| TACE before LT |  |  |  |  |
| Yes | 24 (35.29%) | 14 (43.75%) | 10 (27.78%) | 0.226 |
| No | 44 (64.71%) | 18 (56.25%) | 26 (72.22%) |  |
| RFA before LT |  |  |  | 1.000 |
| Yes | 5 (7.4%) | 3 (8.3%) | 2 (6.3%) |  |
| No | 63 (92.6%) | 33 (91.7%) | 30 (93.8%) |  |
| MELD | 21.47±11.14 | 18.06±9.34 | 24.50±11.83 | ***0.031*** |
| Child-Pugh | 8.78±2.23 | 8.41±2.01 | 9.11±2.38 | 0.126 |
| Capsule |  |  |  | 0.808 |
| Complete | 36 (52.9%) | 20 (55.6%) | 16 (50.0%) |  |
| Incomplete | 32 (47.1%) | 16 (44.4%) | 16 (50.0%) |  |
| HBV |  |  |  | 0.725 |
| + | 59 (86.8%) | 32 (88.9%) | 27 (84.4%) |  |
| - | 9 (13.2%) | 4 (11.1%) | 5 (15.6%) |  |

Table S4 Univariate and multivariate Cox regression analysis of risk factors for OS in recipients with high USP22 expression

| Variable | Univariate analysis | | |  | Multivariate analysis | | | |
| --- | --- | --- | --- | --- | --- | --- | --- | --- |
|  | Exp(B) | 95% CI | *P* | |  | Exp (B) | 95% CI | *P* |
| Use of sirolimus | 0.389 | 0.181-0.836 | ***0.016*** | |  | 0.268 | 0.107-0.671 | ***0.005*** |
| Age | 0.993 | 0.954-1.033 | 0.716 | |  |  |  |  |
| Gender (female) | / |  |  | |  |  |  |  |
| Pathological grade (low) | 3.197 | 1.461-6.998 | ***0.004*** | |  | 3.378 | 1.237-9.225 | ***0.018*** |
| Tumor nodes ≥ 2 | 1.499 | 0.523-4.297 | 0.451 | |  |  |  |  |
| Vascular invasion | 2.895 | 1.286-6.517 | ***0.010*** | |  |  |  | 0.101 |
| Largest tumor size≥ 5cm | 3.956 | 0.539-29.058 | 0.176 | |  |  |  |  |
| AFP ≥ 400 ng/mL | 2.772 | 1.351-5.689 | ***0.005*** | |  |  |  | 0.186 |
| TACE before LT | 0.699 | 0.331-1.477 | 0.348 | |  |  |  |  |
| RFA before LT | 0.341 | 0.046-2.503 | 0.290 | |  |  |  |  |
| MELD | 1.045 | 1.011-1.080 | ***0.009*** | |  |  |  | 0.488 |
| Child-Pugh | 1.376 | 1.124-1.686 | ***0.002*** | |  | 1.496 | 1.108-2.019 | ***0.009*** |
| Capsule (Complete) | 0.496 | 0.235-1.046 | 0.066 | |  |  |  |  |
| HBV | 2.740 | 0.651-11.536 | 0.169 | |  |  |  |  |

Table S5

| gRNA | Sequence |
| --- | --- |
| gRNA1 | AACCGGAAACCTAGTAGTCCAGG |
| gRNA2 | TCCTGGGGGGACAACCCGCAGGG |
| gRNA3 | TCTAATGCACCTGGACTACTAGG |
| gRNA4 | CCTGGGGGGACAACCCGCAGGGG |

Table S6

| Primers | Sequence |
| --- | --- |
| Usp22-F | GGCACTCTGGATCCAAACCTAAT |
| Usp22-R | AACCTCATAGCACTGAATACTCCA |
| Alb-MF | GAAGCAGAAGCTTAGGAAGATGG |
| Alb-MR | TTGGCCCCTTACCATAACTG |

Usp22: Homozygotes: one band with 250 bp

Heterozygotes: two bands with 250 bp and 190 bp

Wildtype allele: one band with 190 bp

Cre amplicon: ~400 bp

Table S7

| Plasmids used in animal experiments | Plasmids used in cells |
| --- | --- |
| pT3-EF1α-c-Myc | pcDNA3.1-Flag-USP22 |
| pT3-EF1α-NRasGV12 | pLVX-Myc-FKBP12 |
| pCMV(CAT)T7-SB100 | pcDNA3.1-Flag-FKBP12 |
| pT3-EF1α-MCS | pLent-Myc-USP22 WT |
| pT3-EF1α-Flag-USP22 | pLent-Myc-USP22 C61,63A |
|  | pLent-myc-USP22 C185A |
|  | pcDNA3.1-HA-Ub |
|  | pcDNA3.1-HA-Ub-K6 |
|  | pcDNA3.1-HA-Ub-K11 |
|  | pcDNA3.1-HA-Ub-K27 |
|  | pcDNA3.1-HA-Ub-K33 |
|  | pcDNA3.1-HA-Ub-K48 |
|  | pcDNA3.1-HA-Ub-K63 |
|  | pLKO.1-shUSP22 |
|  | pLVX-RFP-GFP-LC3 |

Table S8

| siRNA | Sequence |
| --- | --- |
| USP22 siRNA-1 | GAGCTACCAGGAGTCCACAAAG |
| USP22 siRNA-2 | TGTGCCAGGACTACATCTATG |
| USP22 siRNA-3 | GCGAAGGGTACTTGCTGTTCTA |
| USP22 siRNA-4 | GCCTACCTGCTGTAAGATTAUG |
| FKBP12 siRNA-1 | GAGUGCAGGUGGAAACCAUTT |
| FKBP12 siRNA-2 | CAGAGAGCCAAACUGACUATT |
| FKBP12 siRNA-3 | CGUCUUCGAUGUGGAGCUUTT |
| TSC1 siRNA-1 | CGGCTGATGTTGTTAAATA |
| TSC1 siRNA-2 | GTGGCCCT-ATGCTTGTAAA |
| BECN1 siRNA-1 | GCTTGGGTGTCCTCACAATTT |
| BECN1 siRNA-2 | CCCGTGGAATGGAATGAGATT |

Table S9

| Antibodies | Company | Product code |
| --- | --- | --- |
| DYKDDDDK Tag | CST | Cat#8146 |
| Anti-Ki67 | Abcam | Cat#ab15580 |
| Anti-USP22 | Abcam | Cat#ab235923 |
| Anti-USP22 | Abcam | Cat#ab195289 |
| β-actin | ABclonal | Cat#AC026 |
| mTOR | CST | Cat#2983 |
| p-mTOR (S2448) | CST | Cat#5536 |
| p70S6K | CST | Cat#2708 |
| p-p70S6K (T389) | CST | Cat#9234 |
| 4EBP1 | CST | Cat#9644 |
| p-4EBP1 (T37/46) | CST | Cat#2855 |
| Anti-FKBP12 | Abcam | Cat#ab2918 |
| Anti-FKBP12 | Abcam | Cat#ab108420 |
| Anti-Myc tag | Abcam | Cat#ab9106 |
| Anti-Flag M2 | Sigma-Aldrich | Cat#F1804 |
| Raptor | CST | Cat#2280 |
| GbetaL | CST | Cat#3274 |
| Anti-Ubiquitin | Abcam | Cat#ab134953 |
| Rabbit IgG, isotype | Abcam | Cat# ab172730 |
| LC3A/B | CST | Cat#12741 |
| SQSTM1/p62 | Proteintech | Cat#18420-1-AP |
| Beclin-1 | CST | Cat#3495 |
| Anti-Hamartin | Abcam | Cat#ab40872 |
| Goat Anti-Rabbit IgG H&L (HRP) | Abcam | Cat#ab6721 |
| Goat Anti-Mouse IgG H&L (HRP) | Abcam | Cat#ab6789 |

Table S10

| Score | Staining intensity |  | Score | Positive cell proportion |
| --- | --- | --- | --- | --- |
| 0 | Negative |  | 1 | ≤25% |
| 1 | Weak positive |  | 2 | 26%-50% |
| 2 | Moderate positive |  | 3 | 51%-75% |
| 3 | Strong positive |  | 4 | >75% |

**FIGURE LEGENDS**


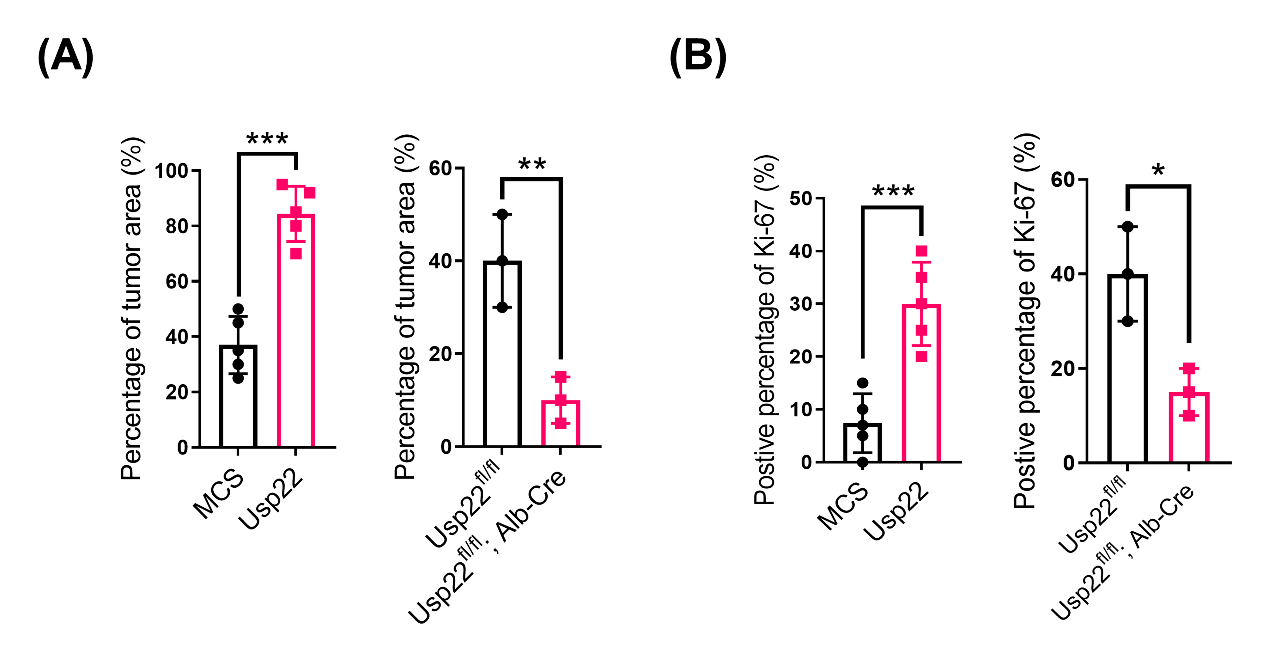


**FIGURE S1** Corresponding quantization diagrams of tumor area and Ki-67 staining. (A) Percentage of tumor area in four groups in Figure 1 D, E. (B) Positive percentage of Ki-67 in four groups in Figure 1 D, E. (**P* < 0.05, ***P* < 0.01, ***P* < 0.001).


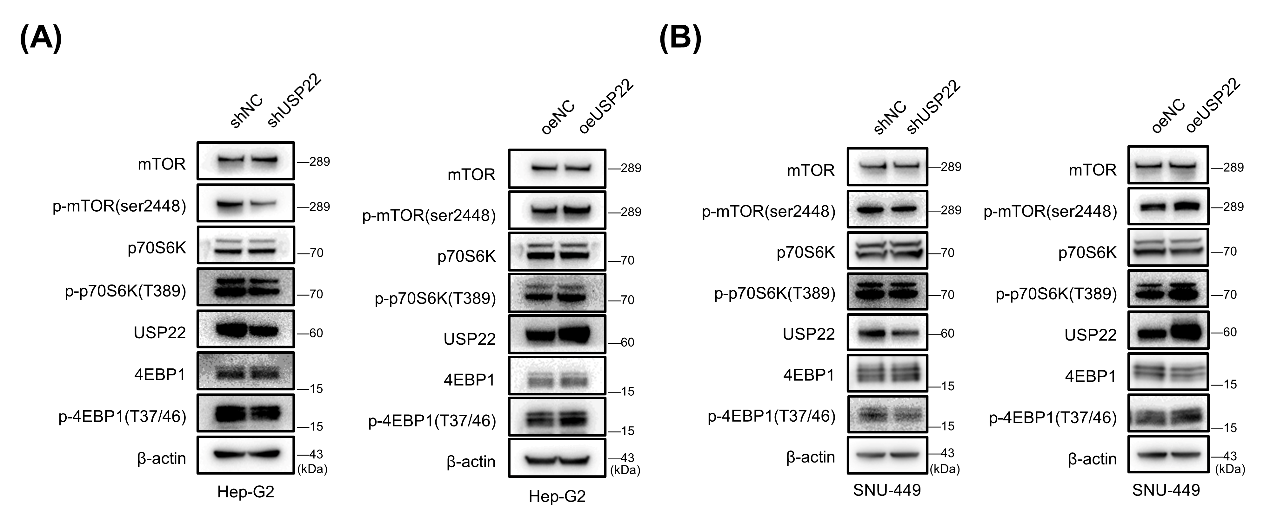


**FIGURE S2** Western blot results of the mTORC1 signaling pathway in HCC cell lines. (A) Western blot results showing key protein expressions of the mTORC1 pathway in Hep-G2 cells. (B) Western blot results showing key protein expressions of the mTORC1 pathway in SNU-449 cells.


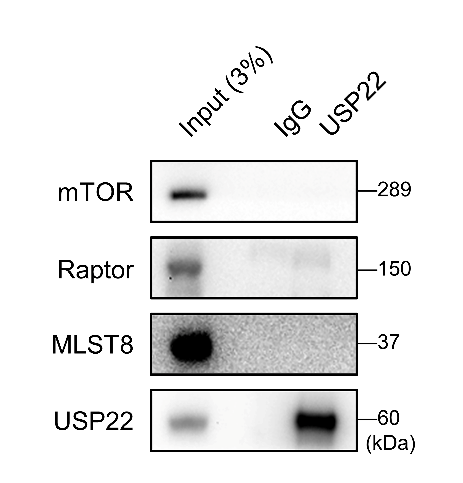


**FIGURE S3** Immunoprecipitation results of USP22 and Raptor, MLST8 or mTOR.


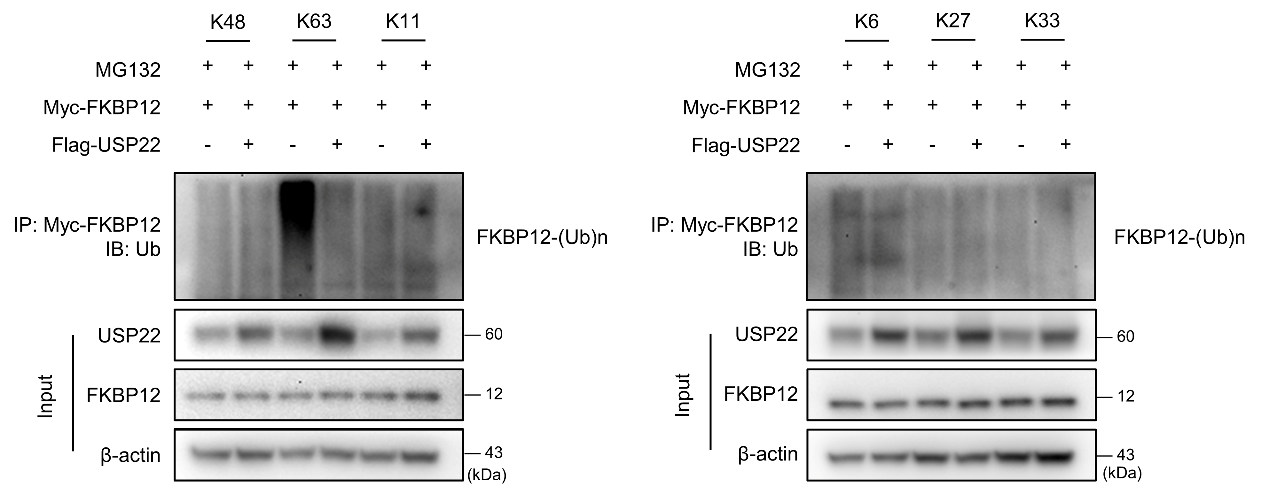


**FIGURE S4** The de-ubiquitination types of USP22 examined using immunoprecipitation and western blotting. Only K63-linked ubiquitination site did not influence FKBP12 poly-ubiquitination.


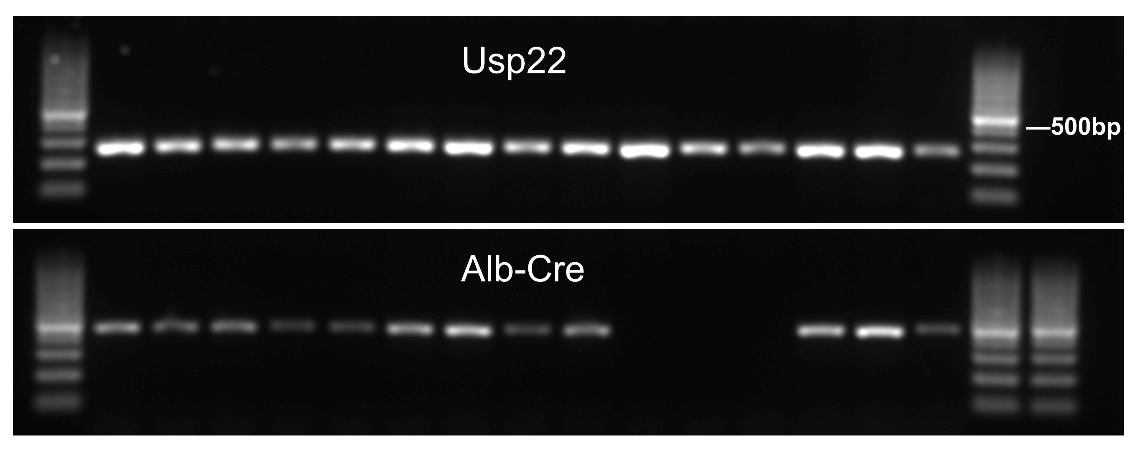


**FIGURE S5** Genotype results of Usp22 Conditional Knockout mouse.


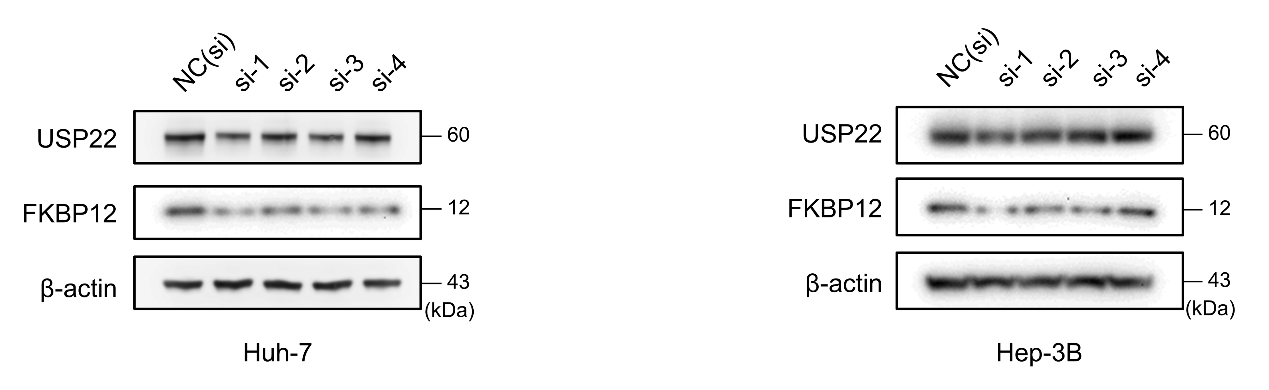


**FIGURE S6** Western blot results of 4 siRNAs targeting USP22 and the corresponding FKBP12 protein levels.
